# “Real-Time” Monitoring of Under-Five Mortality: A Vision Tempered by Reality

**DOI:** 10.1371/journal.pmed.1001912

**Published:** 2016-01-25

**Authors:** Jennifer Bryce, RMM Working Group

**Affiliations:** Institute for International Programs, Johns Hopkins Bloomberg School of Public Health, Baltimore, Maryland, United States of America

## Abstract

Jennifer Bryce and the RMM Working Group describe the origin and rationale of the Real-Time Monitoring of Under-Five Mortality Collection.

**Summary Points**

- Civil registration and vital statistics (CRVS) systems have emerged as a major priority in the post-2015 development agenda, largely based on their assumed potential to generate population data for use in guiding investments and managing programs in low-income countries.
- The evidence base on how best to design, implement, and sustain such systems is weak, especially for mortality statistics. There are global calls for new research and analysis to inform the CRVS agenda, whether the results are positive or negative.
- The Real-Time Monitoring of Under-Five Mortality (RMM) project aimed to develop and test methods for producing estimates of child mortality for recent periods of one year or less. Three broad approaches were assessed: community-based reporting of vital events; calibration of facility data to represent deaths in the population; and rapid survey methods.
- Between 2008 and 2013, we applied and rigorously evaluated at least two of these approaches in each of five countries in sub-Saharan Africa: Ethiopia, Ghana, Malawi, Mali, and Niger.
- The RMM project, reported here as the PLOS Collection on RMM, has generated an important body of evidence that can inform efforts to strengthen CRVS in low-income countries and provides a foundation for continuing CRVS implementation research.

## Introduction

Strengthening civil registration and vital statistics (CVRS) systems in low-income countries is a central component of emerging plans for the post-2015 development agenda [1], and a major investment area for key global health and development funders [2]. A dominant theme in the investment case for CRVS is the potential of such systems to generate valid and timely population data to support sound program planning, management, and evaluation.

Can CRVS systems fulfill this promise and produce population data of adequate quality to support policy and program decisions? Few formal studies have addressed this question, and global leaders have called urgently for new evidence to inform the CRVS agenda [3,4]. The PLOS Collection on Real-Time Monitoring of Under-Five Mortality (RMM) contributes to this process by reporting on a seven-year program of work in five countries in sub-Saharan Africa that aimed to develop and test methods for generating annual estimates of under-five mortality, a key measure for health investments, for recent periods.

This paper introduces readers to the origins and rationale of the RMM project, describes how it was implemented, and outlines the aims of the PLOS Collection on RMM.

## Origins and Rationale

In 2006, the leadership of Canada’s bilateral program in child survival approached the Institute for International Programs (IIP) at Johns Hopkins University with a challenge: How can countries and their development partners measure (not model) rates of under-five mortality in “real time,” either continuously or for periods no longer than three months? The request was the result of a very practical problem–neither Canada nor the governments they were supporting had a means of determining whether their investments were leading to real changes in population health, and thus contributing to achieving the Millennium Development Goal for child survival [5]. At that time, Canada’s experiences with modeled estimates had led to disappointment, as claims of dramatic reductions in under-five mortality had recently been discredited through comparison with estimates measured by household surveys [6]. Going forward, Canada wanted to tie its investments in child survival to measured impact. This commitment to using measured changes in under-five mortality as a key metric in assessing program effectiveness became the hallmark of the Catalytic Initiative to Save a Million Lives, in which Canada was joined by a coalition of other bilateral donors and foundations [7].

Our first response to this request was skepticism. A child death is a relatively rare event the risk of which varies seasonally, and the measurement of such risk requires large sample sizes. Underreporting of neonatal deaths is a continuing and difficult problem, with no easy solutions [8]. Cross-sectional measurements for periods of less than one year are unlikely to produce informative results, even if there were a practical means of implementing them. Health information systems in most countries with high under-five mortality were, and are, too weak to maintain rigorous routine measurement, and in many settings are limited to reporting on deaths among those in contact with the health system. A year of discussion followed, leading to a compromise: IIP would convene a technical consultation, bringing together scientists from a range of disciplines with experience in mortality measurement, to determine whether it was possible to devise a system to address the RMM challenge. If the consultation outcomes were positive, the findings could be used to guide the development of a research program aimed at developing and testing innovative approaches for generating measurements of child mortality in real time, which Canada and other governments and donors could use as triggers to release grant funds to implementing agencies in child survival.

IIP convened the consultation in July 2007. The 19 participants included demographers, epidemiologists, health systems scientists, and economists drawn from academic, research, governmental, and donor agencies, all with experience and expertise in mortality measurement. They presented and discussed measurement approaches, alone and in combination, that included facility-based options, full and sample vital registration systems, a variety of community-based options, and innovative strategies such as those used in emergency settings (e.g., monitoring graveyards). We asked participants to be creative in imagining new approaches that might have untapped potential for the measurement of child mortality at population level.

A full report of the consultation outcomes is available elsewhere [9], but among the recommendations was strong encouragement that IIP move forward to develop and test alternative methods, including innovative approaches based on household surveys, recording of deaths at community level, and using facility death records after adjustment to correct for deaths occurring elsewhere. The group was prescient in calling for the development and improvement of vital registration systems in low-income countries as an important long-term priority, and in anticipating that one of the most important challenges in the RMM work would be obtaining adequate sample sizes to support estimation of significant changes in mortality within short time periods. These and other conclusions of the consultation served as a basis for designing the RMM project.

Canada’s investments under the Catalytic Initiative included the provision of $105 million in funding (2007–2013) to the Integrated Health Systems Strengthening (IHSS) in Africa project, implemented by UNICEF in six sub-Saharan countries [7]. IHSS aimed to train and equip front-line health workers to provide proven and affordable services to children and pregnant women. A major focus was the development and implementation of integrated community case management (ICCM) programs, where health workers deliver services at the community level to combat the three main killers of children younger than the age of five: malaria, diarrhea, and pneumonia. The RMM project was therefore initially designed with the dual objectives of: 1) developing and testing innovative RMM methods; and 2) using those methods to measure the impact of IHSS project activities on under-five mortality, so that the results could serve as a trigger for release of continuation funding.

## The Real-Time Monitoring of Under-Five Mortality Project

Representatives from UNICEF, the Canadian government, and IIP met in March 2008 and selected the initial RMM countries from among those implementing IHSS. Details of the country selection criteria and how the participating countries changed over time are available in S1 Text. The final portfolio of RMM countries covered in this Collection includes Ethiopia, Ghana, Malawi, Mali, and Niger. The health information systems in these five countries are weak, and unable at present to generate recent national or subnational estimates of under-five mortality adequate to support public health decision-making [10].

We conducted a series of assessment and design visits to each RMM priority country, often accompanied by staff from UNICEF and the Canadian International Development Agency (now known as Foreign Affairs, Trade and Development Canada), to assess government interest, identify in-country research partners, and assess opportunities for the implementation and testing of RMM approaches in ways that would reinforce existing vital statistics monitoring in each setting. Formative research studies informed the designs of approaches involving community health workers. At least two methods were tested in each country setting, to increase efficiency. Table 1 provides definitions of terms as used in this work.

**Table 1:** Definitions of terms.

Term	Definition as used in the RMM Collection
Accuracy	The degree to which estimates from a given estimation method are consistent with those of a given validation standard.
Civil registration	The continuous, permanent, compulsory, and universal recording of the occurrence and characteristics of events, including vital events in accordance with the legal requirements of a country.
Completeness	The extent to which a given data collection system documents vital events relative to that of a current best-practice data collection method.
Current best practice	We use this term to refer to methods for measuring vital events or vital rates that represent state-of-the-art practice. We use this in preference to “gold standard” because no measurement technique is perfect.
Real-time	Periods of 12 months or less.
Scale-up	The expansion of implementation from limited geographical areas to full national coverage.
Validation	The comparison of one set of results against another set of results from current best practice to assess the level of error in the former result.
Vital statistics system	The components of a vital statistics system are: a) legal registration; b) statistical reporting; and c) collection, compilation, and dissemination of statistics pertaining to vital events. The vital events of interest are: live births, adoptions, legitimations, and recognitions; deaths and fetal deaths; and marriages, divorces, separations, and annulments of marriage.^i^
Full birth history	Data collected from a woman on (at least) the date of birth, survival status, and age at death if dead, of each live birth she has had.
Full pregnancy history	Data collected from a woman on (at least) the date of completion, survival status, months of gestation for a pregnancy loss or age at death for a live birth that subsequently died, of each pregnancy she has had.
Summary birth history	Data collected from a woman on (at least) her age, number of live births, and number of those births that have died.

^i^ United Nations Department of Economic and Social Affairs, Statistics Division. Principles and Recommendations for a Vital Statistics System. Statistical Papers, Series M No. 19/Rev.3. New York: United Nations; 2014.

By 2010, it was clear that it would not be possible to achieve the second RMM project objective of using proven methods to measure IHSS impact. The timetables for both RMM and IHSS were too short to implement and assess the methods, and then to include those that produced accurate results in evaluation designs that were coordinated with the timing and geographic rollout of IHSS activities. From that point forward the objectives of the project were refocused on assessing the feasibility and accuracy of various methodological approaches.

We tested three broad types of RMM approaches, each adapted to the specific opportunities present at country level (Table 2). The first RMM approach relied on community-based workers to report on the vital events that occurred in their local area. The geographic scope of RMM community-based studies was defined in collaboration with Ministries of Health and IHSS partners, and included several districts or their equivalent in each setting. CRVS advocates have described community-based approaches as underexploited but promising [4], because in an increasing number of countries, community workers are already responsible for vital events reporting and registration [11–13]. We were able to implement locally-adapted RMM methods based on community reporting in all countries except Niger. We assessed the accuracy of each method by comparing the frequency of reported events with those identified through a “best practice” census (in Ghana and Mali) or survey (in Ethiopia and Malawi) [14–17]. The results from Ghana are still under review due to data quality concerns and are not included in the Collection. We obtained ethical approval in each country and from the Johns Hopkins University Bloomberg School of Public Health in Baltimore, Maryland, USA. We presented interim and final results to Steering Committee members in each country and discussed their implications.

**Table 2:** Real-time mortality monitoring (RMM) approaches tested, by country.

	Community-based reporting on vital events		Adjusted health facility data	Rapid household survey methods	
	*Paid worker*	*Unpaid worker*		*Repeated*	*Imputed*
**Ethiopia**	✓			✓	✓
**Ghana**		✓			✓
**Malawi**	✓		✓	✓	✓
**Mali**		✓			✓
**Niger**				✓	✓

The second RMM approach relied on child deaths recorded in health facilities, and calibrated them using other data sources so that they represent all deaths in the population. We felt it was important to test this approach, because the World Health Organization currently recommends that countries with weak CRVS systems should strengthen their capacity for recording deaths and their causes in health facilities [4]. We hypothesized that recording place of death for deaths reported in a representative household survey would provide a measure of the proportion of deaths that occur in facilities, and that the inverse of this proportion could then be applied to recorded facility deaths to estimate total deaths. We selected the two RMM districts in Malawi as the most promising setting in which to test this method, because it had the highest rate of institutional births (69%) among all the study areas at the time RMM was designed [18].

The third RMM approach tested whether the full birth histories collected from women of reproductive age during household survey interviews, which involve many questions and take considerable time, could be replaced by shorter summary birth histories and still produce reasonably accurate estimates of under-five mortality for recent annual periods. If so, information on under-five mortality could be collected as a part of national censuses or other household surveys conducted for various purposes, with little if any added cost. We developed and tested two different methods for doing this, with at least one test in each of the five RMM countries [19].

We tracked the financial costs of implementing each RMM method prospectively, based on standard templates completed by staff of the in-country RMM research organization. The Collection reports on costs for the community-based approaches in each country, both in the synthesis paper [14] and in the individual country papers.

In Malawi, the RMM work served as a platform for additional focused studies of specific methodological issues. Joos and colleagues conducted a trial among the participating RMM community-based workers to determine if frequent, specially-designed text messages would improve the completeness of reporting of pregnancy outcomes [20].

## Aims of the Collection

There are three reasons why we have chosen to publish the core RMM papers together, as a Collection, rather than as individual papers in various journals. Each of these reasons represents a challenge faced by the project, and has served as an important source of learning [21].

First, we want to encourage further analysis of the RMM data, and replications of the RMM results. The RMM findings report on “real-world” field studies conducted in collaboration with government agencies and research institutions in five countries. The time, resources, and commitment required to implement these studies was enormous. It is particularly important, therefore, that the results are reported on fully and transparently, to support replication and comparison with similar efforts that may be undertaken in the future. We have archived, anonymized, and made publicly available the datasets upon which the RMM findings are based. All datasets collected through the RMM project are open access and can be found at http://dx.doi.org/10.7281/T1F769G3 along with their associated documentation. Additional survey datasets used for the calibration, testing, and validation of the RMM rapid survey methods are available through the UNICEF and ICF Macro portals associated with the Multiple Indicator Cluster Surveys program and the Demographic and Health Surveys program [22,23].

Second, the RMM findings demonstrate clearly the importance of publishing negative results as well as positive results, as an essential part of the scientific enterprise [24]. In the case of RMM, there is major global momentum building behind efforts to strengthen CRVS systems, despite the widespread acknowledgment that there is little information available–either from carefully synthesized previous experience or from more formal research studies such as those presented here–as to how this should best be done. We hope that the initial Collection, and periodic additions to it, can serve as a platform for these findings to inform future research, planning, and investment. We encourage readers to check for updates to the RMM Collection (http://www.ploscollections.org/rmm) and to take part in the discussion on the PLOS Collections Blog (http://blogs.plos.org/collections).

Third, we believe that the RMM findings offer a clear message of caution to those who hope and believe that strengthening CRVS systems in low-income countries will be feasible in the short term, and will produce mortality statistics of adequate quality to support sound decision-making. Unless challenges such as those documented by the RMM project are anticipated and incorporated into planning, early efforts to strengthen CRVS systems may fail, endangering those that follow and certainly wasting valuable time and resources.

## Supporting Information

S1 Text Evolution of RMM country portfolio. (PDF)
