# Supplementary material for: “Real-Time” Monitoring of Under-Five Mortality: A Vision Tempered by Reality
Source: PLoS Med. 2016 Jan 25;13(1):e1001912. doi: 10.1371/journal.pmed.1001912 (PMC4726699; doi:10.1371/journal.pmed.1001912)
Supplement: S1 Text — (PDF) [file pmed.1001912.s001.pdf]

S1 Text

Evolution of the portfolio of RMM countries

UNICEF, Canada and IIP representatives met in March 2008 and reviewed each of the seven countries planned for IHSS investment to select a subset for RMM. A central criterion was a high probability of strong IHSS implementation, as reflected in levels of planned project investment and informal assessments of government commitment to the project. Such strong implementation would plausibly lead to swift and measureable reductions in under-five mortality that could be captured by RMM to ensure prompt release of continuation funding from Canada. Consideration was also given to regional diversity, the presence of an in-country research institution capable of collaborating with the RMM team, and the potential for joint funding for a full evaluation of Catalytic Initiative activities from the Bill & Melinda Gates Foundation (Mozambique and Malawi). The initial set of RMM countries included Ethiopia, Ghana, Malawi, Mali and Mozambique.

The priority RMM countries changed over time. UNICEF and Canada proposed dropping Ethiopia in 2009, because initial assessment visits revealed that the national policy – and therefore IHSS project plans - did not include community management of pneumonia, the illness with the highest burden among children under-five in the country. In 2010, the MOH adopted a policy supporting integrated community case management (iCCM), which included administration of antibiotics for pneumonia at community level, and Ethiopia was reinstated as a priority RMM country. Mozambique was also one of the original RMM priority countries, but Catalytic Initiative donors and UN partners recommended it be dropped in 2010 because program activities focused on maternal health and were unlikely to result in measureable reductions in under-five mortality by 2012. Niger was not originally identified as an RMM priority country, but in 2009 UNICEF compiled the results of several nationally-representative household surveys showing dramatic increases in coverage for high-impact child survival interventions, and with agreement

from Canada request that it be considered for inclusion in RMM. The final portfolio of RMM countries reflected in this Collection includes Ethiopia, Ghana, Malawi, Mali and Niger.
